# Rejection Retention as a Measure of Chlorine Resistance: Initial-Rejection Bias in Filler-Modified Poly(vinyl alcohol) Reverse Osmosis Membranes

**DOI:** 10.3390/polym18212796

**Published:** 2026-11-09

**Authors:** Wail Falath

**Affiliations:** 1 Department of Materials Science and Engineering, King Fahd University of Petroleum & Minerals, Dhahran 31261, Saudi Arabia; wfallata@kfupm.edu.sa; 2 Center of Research Excellence in Desalination and Water Treatment, King Fahd University of Petroleum & Minerals, Dhahran 31261, Saudi Arabia

**Keywords:** crosslinked poly(vinyl alcohol), reverse osmosis, chlorine resistance, salt passage, rejection retention, nanocomposite membranes

## Abstract

Chlorine resistance of reverse osmosis membranes is usually reported as the fraction of salt rejection retained after chlorination, a metric that depends on the initial rejection. This secondary reanalysis quantifies the dependence for published data on substrate-free poly(vinyl alcohol) (PVA) membranes containing multi-walled carbon nanotubes (MWCNT), Gum Arabic (GA) or graphene oxide (GO). Within the solution–diffusion model, the change in salt passage was expressed as an observed ratio, k_R_, which also includes any change in flux or swelling and is not a direct measure of chemical degradation. Judged by rejection retention, all three fillers reduced the relative rejection loss about tenfold at their best-performing loadings. Relative to each study’s control, however, GO produced no consistent reduction in k_R_ across loadings (3.11 ± 0.48 versus 3.20); MWCNT produced a moderate reduction (1.16–1.49 versus 1.76) and GA a reduction only at 0.1–0.5 wt%; these differences persisted, marginally for MWCNT, at rejection uncertainties of two percentage points. For GO and GA, most or all of the apparent improvement (97–111% in the more robust decomposition order) reflected the higher initial rejection. Rejection retention therefore overstates the benefit of fillers that raise selectivity; the salt-passage ratio offers a simple correction when post-exposure flux is unavailable.

## 1. Introduction

Reverse osmosis (RO) has become the dominant technology for seawater and brackish water desalination [1,2], and the performance of an RO membrane is governed predominantly by its thin selective layer [3,4]. This layer is almost always an aromatic polyamide, which is attacked by free chlorine through N-chlorination and ring chlorination and deteriorates markedly after exposures of the order of 1000 ppm·h [5,6,7,8]. Feed water is therefore dechlorinated upstream, which in turn exposes the membranes to biofouling [9,10,11]. Proposed remedies include sacrificial coatings [12,13], selective layers without amide linkages [14] and fillers in the selective layer. Carbon nanotubes, which are also antibacterial [15,16], are one example: a polyamide membrane with a high nanotube loading retained its rejection after an exposure that degraded the nanotube-free membrane [17,18]. Graphene oxide (GO) has attracted similar interest [19,20,21]. Chlorination of polyamide consumes hydrogen-bonding sites [22] and depends on pH and dose [23], and claims of improved stability are most convincing when several lines of evidence agree, as in a recent study combining molecular simulation with prolonged exposure [24].

Poly(vinyl alcohol) (PVA) is an attractive alternative selective material: it is hydrophilic, readily film-forming and contains none of the amide groups responsible for the chlorine sensitivity of polyamide [25,26]. Because its transport properties are governed by the density of a crosslinked, water-swollen network, fillers can alter selectivity not only through their own presence but also by modifying the structure of that network, for example, through hydrogen bonding with PVA [25,27]. In previous work, a substrate-free PVA active layer was prepared by crosslinking PVA with bisphenol A diglycidyl ether (DGEBA) in the presence of Pluronic F-127 as a pore former, and was subsequently modified with multi-walled carbon nanotubes (MWCNT) [28], Gum Arabic (GA) [29] and GO [30]. In each case, the modified membranes lost markedly less rejection on chlorination than the unmodified membrane. The GA study attributed this improvement to hydrogen bonding between PVA and Gum Arabic [29], and the MWCNT study to physical crosslinking by the nanotubes, which was thought to reduce the sites available for chlorine attack [28]; the GO study did not propose a specific mechanism [30].

Those studies, in common with most of the literature, expressed chlorine resistance as the percentage of initial rejection retained after exposure. Although convenient, this metric is not independent of the initial rejection. Within the solution–diffusion model, salt passage is governed by the ratio of the salt permeance B (often termed the salt permeability coefficient) to the water flux [31,32]. Water and salt permeances, rather than flux and rejection, characterize the intrinsic transport properties of a membrane [33,34], and the trade-off between permeability and selectivity in polymeric membranes is expressed in the same terms [35,36]; for desalination, a gain in selectivity has been argued to be more valuable than a gain in water permeability [37]. A membrane that transmits little salt therefore shows only a small loss of rejection even when its salt permeance rises considerably, so a filler that increases selectivity will also appear to increase chlorine resistance. Salt passage rather than rejection is already used to normalize RO plant performance data [38], and the ratio of salt permeability before and after chlorination has been adopted in the patent literature [39]. To the author’s knowledge, however, the size of this bias in published chlorine-resistance data has not been quantified, although it bears directly on the many reports of chlorine-resistant nanocomposite membranes.

The three PVA studies constitute a well-suited data set for addressing this question. They employed nominally similar formulations, an identical casting route and test cell, and a comparable chlorination protocol, each across a complete series of filler loadings, which limits much of the variability that confounds inter-laboratory comparisons [40], although batch-to-batch and inter-study differences remain. Because all three studies were carried out in the same laboratory, they also share systematic features, and they cannot be treated as independent replications. The present work is a secondary reanalysis of these published series; no new experiments were performed, and no new membrane is proposed. Its contribution is methodological: it quantifies the dependence of rejection retention on the initial rejection and distinguishes the apparent chlorine resistance indicated by this metric from the observed change in salt passage. An observed salt-passage ratio, k_R_, is determined for every membrane in the three series, and a sequential counterfactual decomposition is used to apportion the apparent benefit of each filler between the higher initial rejection and a reduction in k_R_. The trends in k_R_, together with the mechanical and surface properties reported in the same studies, are then examined for consistency with the protection mechanisms proposed for the three fillers.

## 2. Materials and Methods

### 2.1. Membranes and Chlorination Data

The data come from three related experimental series on nominally similar substrate-free PVA membranes [28,29,30] (Table 1). In all three studies, filler loadings were given as weight percentages of the casting solution, which consisted of PVA in dimethyl sulfoxide (17:83 by weight), 0.16 wt% DGEBA and 6 wt% Pluronic F-127 [28,29,30]. The loadings are therefore on a common basis, although the filler fractions in the dry film are higher. The membranes were cast to a nominal thickness of 0.1 mm and tested in a dead-end stirred cell (Sterlitech HP4750, 14.6 cm^2^); the performance tests were run at 5.52 MPa (55.2 bar).

The chlorination protocol was essentially the same in the three studies. NaCl rejection was first measured with a 2000 mg L^−1^ NaCl feed. The membrane was then removed from the cell, rinsed with distilled water and immersed for 2 h in a 2000 mg L^−1^ hypochlorite solution, prepared by diluting a commercial NaClO solution (10 wt% free chlorine, as stated in [28,29]) with distilled water and adjusted to pH 4.0 with 0.1 M HCl. Exposure was at ambient temperature in all three studies (stated as 25 °C in two of them). The membrane was then rinsed thoroughly, returned to the cell and retested with 2000 mg L^−1^ NaCl. The exposure is described here by the parameters of the source protocol, which is a nominal hypochlorite concentration of 2000 mg L^−1^ for 2 h (about 4000 mg L^−1^·h); the analysis does not depend on further details such as the free-chlorine basis of the concentration, the solution volume or the residual chlorine. In two of the studies, water flux was also measured after exposure [28,29], but only the rejections were reported, so the present analysis is based on rejection alone. Because the modified membranes and the control of each study were chlorinated under the same protocol, each modified membrane is compared only with the control of its own study (Section 2.4).

The rejections before and after chlorination for every composition are listed in Table 2. All values were taken from the original laboratory records of the three studies and agree, within rounding, with those reported in the published papers; Table S1 lists them with the corresponding source publications.

The source studies also report, for every composition, the water contact angle, the surface roughness (Ra and Rq) from atomic force microscopy, Young’s modulus from tensile tests, and the water flux and salt rejection at 5.52 MPa with a 3.28 wt% natural sea-salt feed [28,29,30]. The values used here (Table S2) were taken from the original laboratory records and agree with the values stated in the source texts to within about one percentage point, the texts quoting rounded values, including the increases in modulus of 80% for GA and more than 100% for GO [29,30] and the maximum flux of 92 L m^−2^ h^−1^ for GO [30]. Table S2 also gives, for each entry, the figure and page of the source publication and the number and type of replicates (technical readings on one membrane, or independently prepared membranes); the analysis uses the mean values. The rejections in the performance tests agree with the before-chlorination rejections in Table 2 to within about one percentage point. The unmodified membranes of the three studies have the same flux (66 L m^−2^ h^−1^) and very similar contact angle, roughness and modulus, but different rejections (about 55% in [28] and 69% in [29,30]).

### 2.2. Rejection and Salt Permeance

Water and salt transport through the selective layer were described by the solution–diffusion model [31,32,41]:

(1) *J_w_* = *A* (Δ*P −* Δ*π*)

(2) *J_s_* = *B* (*C_m_ − C_p_*)

where J_w_ and J_s_ are the water and salt fluxes, ΔP is the applied pressure, Δπ = π(C_m_) − π(C_p_) is the transmembrane osmotic pressure difference, and C_m_ and C_p_ are the salt concentrations at the feed-side membrane surface and in the permeate, respectively. Since C_p_ = J_s_/J_w_, Equation (2) yields C_p_/C_m_ = B/(J_w_ + B). Neglecting concentration polarization, C_m_ equals the bulk feed concentration, and the observed rejection R becomes

(3) *R* = *J_w_*/(*J_w_ + B*)*, so that* (1 *− R*)/*R* = *B*/*J_w_*

Equation (3) is exact within the model and does not require the permeate concentration to be small. When concentration polarization is taken into account, the film model gives (1 − R)/R = (B/J_w_) exp(J_w_/k_m_), where k_m_ is the feed-side mass-transfer coefficient [42,43].

### 2.3. Observed Salt-Passage Ratio

The effect of chlorination was quantified by an observed salt-passage ratio, k_R_, defined directly in terms of the rejections measured before (R_b_) and after (R_a_) exposure:

(4) *k_R_* = [(1 *− R_a_*)/*R_a_*]/[(1 *− R_b_*)/*R_b_*]

A value of k_R_ = 1 indicates that B/J_w_, and hence the rejection, was unaffected by chlorination. By Equation (3), k_R_ is equal to (B/J_w_)_after_/(B/J_w_)_before_ only when concentration polarization is negligible; otherwise, the film model gives k_R_ = [(B/J_w_)_after_/(B/J_w_)_before_] × exp[(J_w,a_ − J_w,b_)/k_m_], where the subscripts a and b denote values after and before exposure, so that k_R_ also reflects any change in water flux and the mass-transfer conditions in the cell. In addition, the change in B itself equals (B/J_w_)_after_/(B/J_w_)_before_ multiplied by J_w,a_/J_w,b_, so that changes in flux or swelling upon chlorination are also embedded in k_R_. Combining both effects gives k_R_ = (B_a_/B_b_)(J_w,b_/J_w,a_) exp[(J_w,a_ − J_w,b_)/k_m_], assuming that k_m_ is unchanged. A filler could therefore alter k_R_ by changing the response of the flux to chlorination, even if the rate of chemical attack on the network were unchanged. As the present analysis uses only the rejections measured before and after exposure, these contributions cannot be separated. Accordingly, k_R_ is not a direct measure of chemical degradation but of the observed chlorination-induced change in salt passage under the stated test conditions, and the term “damage” is used in this sense throughout. Because (1 − R)/R approaches the salt passage, 1 − R, when the rejection is high, k_R_ is referred to as a salt-passage ratio; strictly, it is a ratio of B/J_w_ values, and “change in salt passage” is used below in this sense. Rearrangement of Equation (4) yields the relative rejection loss D produced by a given k_R_ from an initial rejection R_b_:

(5) *D* = 1 *− R_a_*/*R_b_* = (*k_R_ −* 1)(1 *− R_b_*)/[*R_b_ + k_R_*(1 *− R_b_*)]

As R_b_ approaches unity, D tends to zero for any finite k_R_. The same change in salt passage therefore always produces a smaller loss of rejection when the initial rejection is higher.

### 2.4. Separating the Effect of Initial Rejection

Each modified membrane was compared with the unmodified membrane of its own study. The three unmodified membranes have nominally the same composition and report the same flux and very similar surface and mechanical properties (Table S2), but their rejections before or after exposure differ, and so do their k_R_ values (1.76, 2.03 and 3.20). Comparing each membrane with the control reported in the same study limits the influence of such differences, although it cannot remove differences in batch, operator or procedure. Since each series has a single control, a batch difference in that control would shift every comparison within the series. The spread among the three controls, a factor of 1.8 in k_R_, shows that such differences can be as large as the effects examined below.

The improvement in each modified membrane relative to its control, D_0_ − D, was partitioned by a sequential counterfactual decomposition. In order A, the initial rejection is varied first. D* is the rejection loss calculated from Equation (5) with the membrane’s own R_b_ and the k_R_ of its control. The difference D_0_ − D* is the improvement due to the higher initial rejection alone, and the remainder, D* − D, is attributed to the reduction in k_R_. In order B, k_R_ is varied first, and the contribution of the initial rejection is evaluated at the membrane’s own k_R_. Because Equation (5) is non-linear, the two orders give different partitions, and both are reported. The share attributable to the initial rejection is the corresponding contribution divided by D_0_ − D; a share above 100% means that the modified membrane had a higher k_R_ than its control, and a negative share means that its initial rejection was lower than that of the control. These shares are model-based attributions under the specified decomposition rather than uniquely identified causal percentages. As an additional sensitivity check, order A was repeated with a pooled control defined as the mean of the three unmodified membranes (R_b_ = 64.0%, R_a_ = 44.4%, k_R_ = 2.22).

### 2.5. Uncertainty and Statistics

The source studies report the performance-test rejections as means of at least three samples [28,29,30], and the chlorination rejections used here are likewise mean values from the laboratory records. As a base case, an uncertainty of ±0.5 percentage points was assigned to each rejection, corresponding to the rounding of the GO records to whole percentages; the other rejections were recorded to 0.1 percentage point. The 95% intervals for k_R_ were obtained by Monte Carlo sampling (20,000 draws), with R_b_ and R_a_ sampled independently from uniform distributions spanning ±0.5 percentage points about the recorded values, all of which lie well within 0–100%. These intervals describe the propagation of the assumed uncertainty and should not be interpreted as replicate-based confidence intervals. Because ±0.5 percentage points may understate the variation between independently prepared membranes, the calculation was repeated with each mean rejection drawn from a normal distribution, truncated to 0–100%, whose standard deviation represents the uncertainty of the mean (its standard error); values of 1 and 2 percentage points were used, and the control rejections were perturbed in the same way (Table S3). R_b_ and R_a_ were drawn independently. This is conservative if the same membranes were tested before and after exposure, because errors common to both measurements would partly cancel in k_R_. For each modified membrane and for the mean of each series, the fraction of draws in which k_R_ was lower than that of the control was recorded. This fraction, referred to below as the probability that k_R_ lies below the control, measures how robust the ordering is to the assumed uncertainty; it is not a formal significance level. The standard deviation was then raised in steps of 0.25 up to 4 percentage points to find the largest value at which this probability remained at least 0.95 for the series mean (Table S4). Because the GA membranes were grouped as 0.1–0.5 and 0.7–0.9 wt% after inspection of the data, and three series were compared, these probabilities are indicative rather than formal. For comparison, the error bars of the performance tests in the source studies indicate standard deviations of mostly 1–3 percentage points between replicate samples [28,29,30], corresponding to standard errors of about 0.6–1.7 percentage points for means of three membranes. This variability is used only as a guide to that of the chlorination tests, which used the same casting route and test cell. Trends with loading were assessed with the Spearman rank correlation coefficient, ρ, using exact two-sided permutation *p*-values, as each series comprises only five modified membranes; given the small sample size and the absence of correction for multiple comparisons, these tests are exploratory.

### 2.6. Property Correlations and Micromechanical Estimate

For each modified membrane, the gain in selectivity relative to its control was expressed as the fold reduction in B/J_w_ in the performance tests, (B/J_w_)_0_/(B/J_w_), obtained from Equation (3) with the rejections at 5.52 MPa. Young’s modulus and k_R_ were likewise expressed relative to the control (E/E_0_ and k_R_/k_R_,0). Spearman rank correlations between these ratios, and with the contact angle and roughness, were computed over the fifteen modified membranes, with two-sided *p*-values from 100,000 random permutations; within-series correlations used exact permutation *p*-values. The selectivity gain and k_R_ both depend on the salt passage of the membrane before chlorination, measured in the performance and chlorination tests, respectively; because these two measurements agree to within one percentage point, a positive correlation between the two ratios is partly a consequence of this common term and is interpreted accordingly.

To assess whether the observed stiffening can be attributed to the fillers acting as rigid reinforcements, the modulus of the composite was estimated with the Halpin–Tsai equations [44]:

(6) *E_c_*/*E_m_* = (1 *+ ζηφ*)/(1 *− ηφ*)*, η* = (*E_f_*/*E_m_ −* 1)/(*E_f_*/*E_m_ + ζ*)

where E_c_, E_m_ and E_f_ are the moduli of the composite, matrix and filler, φ is the filler volume fraction and ζ is a shape factor, taken as 2α/3 for platelets of aspect ratio α and as 2l/d for tubes of length l and diameter d. Equation (6) gives the modulus parallel to perfectly aligned fillers (along the tube axis or in the platelet plane), denoted E_11_; the modulus perpendicular to the fillers, E_22_, is taken as approximately equal to E_m_, since fillers contribute little stiffness in that direction. In solution-cast films, GO platelets tend to lie in the plane of the film, so the in-plane tensile modulus approaches the aligned value E_11_; random three-dimensional orientation, E ≈ 0.49E_11_ + 0.51E_22_ [45], was calculated for comparison. Nanotubes were treated as randomly oriented in the plane of the film, E ≈ (3/8)E_11_ + (5/8)E_22_ [46], with the aligned value as an upper bound. The matrix modulus was taken as 0.2 GPa, the modulus of the controls (190–200 MPa). The filler volume fraction in the dry film was obtained from the loading in the casting solution, taking the PVA solution as 17 g of PVA per 100 g with the additive loadings on the same basis, for two limiting cases: all solids (PVA, Pluronic F-127, DGEBA and filler) retained in the film, and Pluronic F-127, an extractable pore former, fully removed with complete densification of the film, using densities of 1.25 g cm^−3^ for the matrix, 1.9 g cm^−3^ for GO and 1.8 g cm^−3^ for MWCNT. As the effective moduli and aspect ratios of the dispersed fillers are uncertain, bounding values were used: E_f_ = 40–250 GPa and α = 300–700 for GO, from the flake size of 0.3–0.7 μm given in [30] and a sheet thickness of about 1 nm, and E_f_ = 100–1000 GPa and l/d = 300–833 for MWCNT, the upper value corresponding to the 12 nm × 10 μm tubes used in [28]. The approach does not apply to Gum Arabic, a soluble polysaccharide conjugated into the network rather than a rigid filler. The resulting ranges are illustrative sensitivity estimates that depend on the assumed morphology, orientation and filler properties; they indicate what rigid reinforcement could plausibly achieve and do not constitute experimental evidence for any particular network structure.

### 2.7. Software and Use of Generative AI

All calculations, including the exact permutation tests and both decomposition orders, were performed in Python 3.12 using NumPy 2.4, SciPy 1.17 and pandas 3.0, and the figures and graphical abstract were generated with Matplotlib 3.10. The complete calculation script, which reproduces Table 2, the property correlations, Table 3 and the sensitivity analyses of Tables S3 and S4, is provided as Code S1; the rejection data are given in Table S1 and the membrane properties in Table S2. Generative AI (Claude Opus 5.5, Anthropic, San Francisco, CA, USA) assisted with literature searching, editing of the manuscript, development of the calculation and plotting scripts, and checking of the calculations. All rejection and property values used in the analysis were taken by the author from the original laboratory records, and the author checked and approved all calculations, figures, interpretations and conclusions.

## 3. Results

### 3.1. Chlorination Data

Table 2 lists the rejections before and after chlorination for all eighteen membranes, together with D, k_R_ and the share of each improvement attributed to the initial rejection under both decomposition orders. Judged by D, all three fillers were highly effective: between the control and the best-performing loading, the relative rejection loss decreased from 25.9% to 2.2% for MWCNT, from 24.2% to 3.1% for GA and from 40.6% to 3.1% for GO, an 8- to 13-fold reduction. The k_R_ values are much less uniform.

### 3.2. Salt-Passage Ratio and Filler Loading

**Figure 1:**
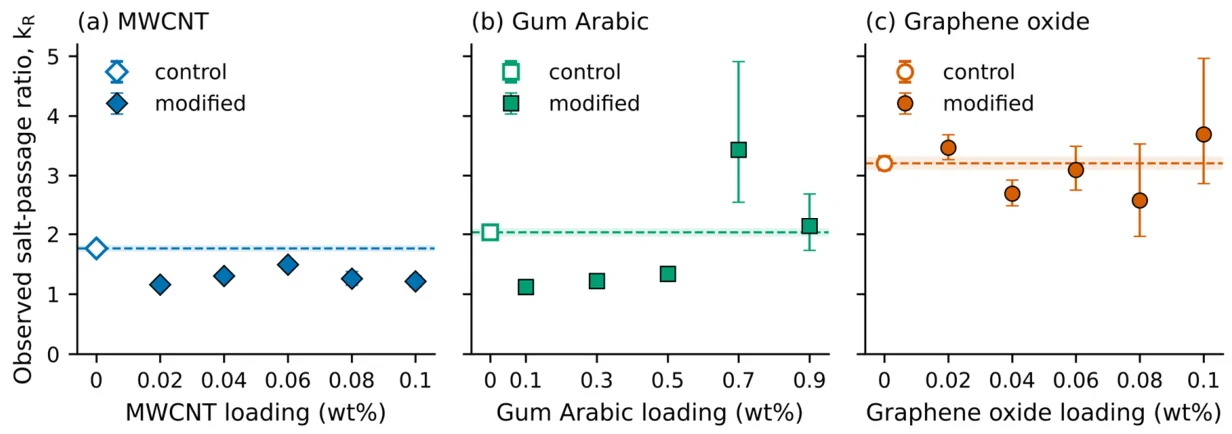
Observed salt-passage ratio k_R_ as a function of filler loading for (**a**) MWCNT, (**b**) Gum Arabic and (**c**) graphene oxide. Open symbols and dashed lines show the control (unmodified membrane) of each study, and the shaded band shows its 95% interval. Error bars are 95% intervals propagated from an assumed ±0.5 percentage point uncertainty in the rejections; the effect of larger uncertainties is given in Tables S3 and S4.

Figure 1 presents k_R_ as a function of filler loading for the three series, with the control of each study shown for reference. As noted in Section 2.3, a difference in k_R_ between a modified membrane and its control is a difference in the observed change in salt passage, which may include a contribution from the response of flux or swelling to chlorination.

For MWCNT (Figure 1a), all five modified membranes exhibit a lower k_R_ than their control (1.16–1.49 versus 1.76); under the base uncertainty, each lies below the control with a probability above 0.99. The mean, 1.29 ± 0.13, lies some 27% below the control value. The reduction is already present at 0.02 wt%, and k_R_ shows no monotonic trend with loading (Spearman ρ = 0.10, exact *p* = 0.950), varying between 1.16 and 1.49; with five points, however, this test has little power. A protective effect of carbon nanotubes has also been reported for polyamide membranes, under different exposure conditions [18].

For GA (Figure 1b), k_R_ falls to 1.12–1.34 at 0.1–0.5 wt%, well below the control value of 2.03, but is 3.43 and 2.14 at 0.7 and 0.9 wt%, no lower than the control. The rank correlation among the modified membranes (ρ = 0.90, exact *p* = 0.083) suggests that k_R_ increases with loading, but it is not statistically significant; with only five points, and with initial rejections of 97–98% at the two highest loadings, where k_R_ is poorly constrained, the trend is tentative. Even so, the low-loading GA membranes underwent a smaller change in salt passage than the high-loading membranes, although the latter lost a similar or smaller fraction of their rejection.

For GO (Figure 1c), the modified membranes yield k_R_ = 3.11 ± 0.48, compared with 3.20 for the control. Two values (2.70 and 2.58) fall below the control and three (3.47, 3.09 and 3.69) lie close to or above it, with no monotonic trend with loading (ρ = 0.10, exact *p* = 0.950). Only the value at 0.04 wt% lies clearly below the control, so GO produced no consistent reduction in k_R_ across loadings.

To test whether these conclusions depend on the assumed uncertainty, the comparisons were repeated with larger uncertainties (Section 2.5). The mean k_R_ of the 0.1–0.5 wt% GA membranes remained below that of the control with a probability of at least 0.95 for standard deviations of up to 3.25 percentage points in each mean rejection (Table S4); at 2 percentage points, it was 0.61 times the control value (95% interval 0.45–0.84; Table S3). For MWCNT, the corresponding limit was 2 percentage points. At that level, the series mean was 0.75 times the control, but the two-sided 95% interval of this ratio (0.55–1.03) just included unity, so the MWCNT reduction is marginal at this uncertainty; individually, only the membrane at 0.02 wt% still lay below the control with a probability above 0.95 (0.99), the others giving 0.77–0.94. For GO, the probability that the series mean lay below the control remained between 0.43 and 0.66 under all the uncertainty assumptions examined (0.43–0.56 in the scan of Table S4), so a larger uncertainty does not reveal a reduction. The same conclusions were obtained when the geometric mean or the median of each series was used in place of the arithmetic mean.

The three controls have nominally identical composition and report the same flux, yet their rejections before or after chlorination, and hence their k_R_ values (1.76, 2.03 and 3.20), differ. This spread is the reason each filler is compared exclusively with its own control.

### 3.3. Contribution of Initial Rejection to the Apparent Improvement

**Figure 2:**
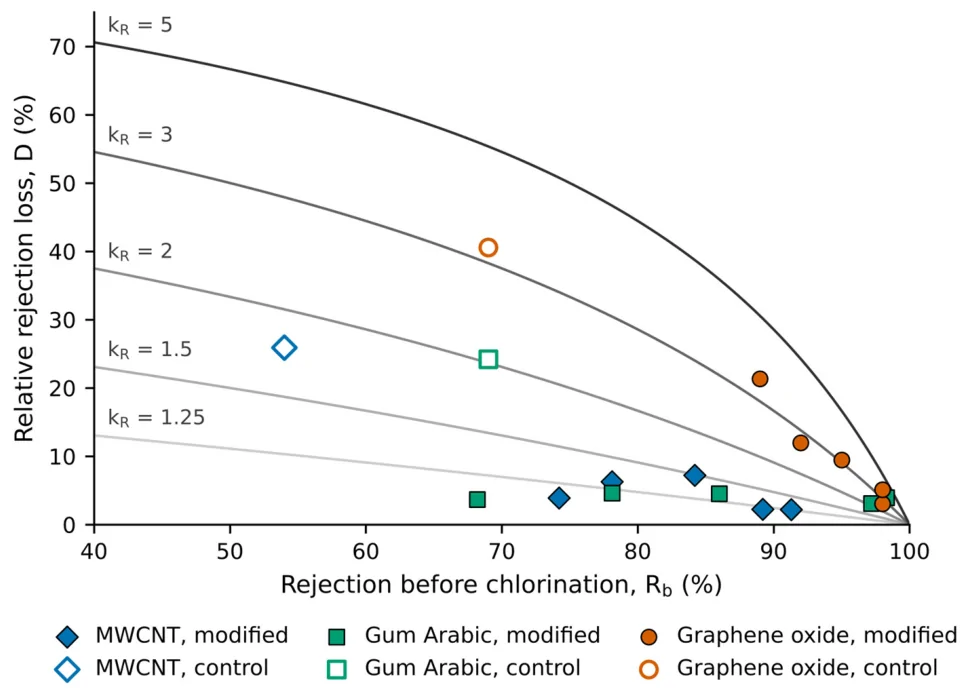
Relative rejection loss D plotted against the rejection before chlorination R_b_ for all membranes. The gray lines are lines of constant salt-passage ratio k_R_ calculated from Equation (5). Open symbols: controls.

Figure 2 plots D against R_b_ for all membranes, together with curves of constant k_R_ calculated from Equation (5). Along each curve, D decreases steeply with increasing initial rejection, even though k_R_, the relative change in B/J_w_, is the same. For k_R_ = 3, for example, D is 38% at an initial rejection of 69% but only 4% at 98%. The GO membranes and their control all lie close to the k_R_ = 3 curve, so the decrease in D observed for GO is largely accounted for by its displacement along that curve. The MWCNT and low-loading GA membranes, in contrast, lie below the curves passing through their respective controls, indicating a reduction in k_R_.

**Figure 3:**
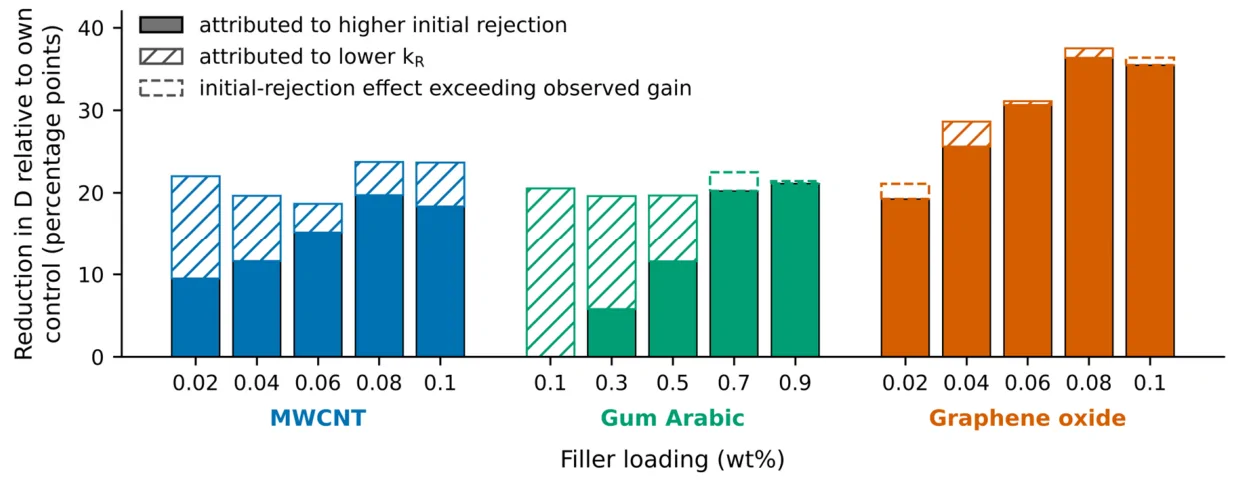
Reduction in the relative rejection loss of each modified membrane compared with the control of its own study, divided under decomposition order A (Section 2.4) into the part attributed to the higher initial rejection (filled) and the part attributed to a lower salt-passage ratio (hatched). Dashed outlines mark cases where the effect of the higher initial rejection exceeds the observed improvement, that is, where k_R_ was higher than for the control.

Figure 3 shows the decomposition of Section 2.4 for order A, and Table 2 gives the shares under both orders. For the MWCNT membrane at 0.08 wt% (R_b_ = 91.3%, D = 2.2%), for example, the k_R_ of its control (1.76) would have produced a rejection loss of D* = 6.2%. Under order A, 19.7 of the 23.7 percentage points of improvement (83%) are therefore attributed to the higher initial rejection and 4.0 points to the reduction in k_R_; under order B, the corresponding share is 35%.

For GO, the share attributed to the initial rejection ranges from 79% to 114% at every loading under either order, as expected from the absence of any systematic change in k_R_. For GA, the improvement at 0.1 and 0.3 wt% arises mainly from a reduction in k_R_, the share attributed to the initial rejection being −2% to 30% under either order; at 0.5 wt%, the attribution depends on the order (59% under order A and 25% under order B), and at 0.7 and 0.9 wt% the share reaches 101–193%. For MWCNT, the attribution depends strongly on the order, ranging from 13 to 60% under order B to 43–83% under order A. At the best-performing loadings identified in the original studies (0.08–0.1 wt% for GO and MWCNT; 0.7–0.9 wt% for GA, where 0.7 wt% gave the highest rejection and 0.9 wt% was identified as optimal [29]), the higher initial rejection thus accounts for most or all of the apparent improvement for GO and GA (97–111% under order A; 79–193% across both orders, order B being poorly constrained at these loadings). For MWCNT, the attributed share ranges from 28% to 83%, depending on the order. With the pooled control, order A yields 67–111% at these loadings. When every rejection was assigned a standard deviation of 2 percentage points, the lower limit of the 95% interval of the order-A share at the best-performing loadings of GO and GA was 67%. Order-B shares at these loadings, where R_b_ is 97–98%, became poorly constrained (Table S3).

### 3.4. Relation to Mechanical and Surface Properties

The mechanical and surface data reported in the source studies were examined for consistency with the trends in k_R_. Because they cover fifteen membranes from a single laboratory, the correlations, shown in Figure S1, are treated as exploratory. Table 3 compares the observed stiffening with the Halpin–Tsai estimates. Across the fifteen modified membranes, the selectivity gain increases with the relative modulus (ρ = 0.66, *p* = 0.009; Figure S1a). The correlation is positive within each series (GO: ρ = 0.97, exact *p* = 0.033; GA: ρ = 0.90, exact *p* = 0.083; MWCNT: ρ = 0.70, exact *p* = 0.233), although only the GO correlation reaches *p* < 0.05, without correction for multiple comparisons. The MWCNT membranes reached the highest relative stiffness (2.25–2.30) with 4- to 8-fold selectivity gains, whereas GO and GA reached 20- to 24-fold gains at 1.8- to 2.0-fold stiffness. The correlation does not establish a causal link. The stiffening may arise from additional physical crosslinking, from a denser hydrogen-bond network or from reinforcement by the filler, and the modulus data cannot distinguish between these.

The selectivity gain was not accompanied by a smaller change in salt passage. Its correlation with the relative k_R_ was positive (ρ = 0.76, *p* = 0.001; Figure S1b), although part of this correlation arises from the before-exposure salt passage on which both quantities depend (Section 2.6), and three of the four membranes with k_R_ above that of their control are among the four most selective. Neither the contact angle nor the roughness was related to k_R_ (ρ = −0.10, *p* = 0.72 for the contact angle, Figure S1c; ρ = −0.04, *p* = 0.88 for the roughness) or to the selectivity gain (ρ = −0.27, *p* = 0.34 and ρ = −0.35, *p* = 0.20).

At the loadings of maximum stiffness, GO and MWCNT raised the modulus 2.02-fold and 2.30-fold, respectively (Table 3). For GO platelets lying in the film plane, the Halpin–Tsai estimate spans 1.23–2.04-fold; the observed value is reached only at the upper limit, which requires the highest filler modulus and aspect ratio together with full extraction of Pluronic F-127 and complete densification of the film. For MWCNT oriented randomly in the film plane, the estimate (1.18–2.14-fold) falls short of the observed 2.30-fold over the whole range of tube properties and Pluronic retention considered, although the aligned limit (1.49–4.03-fold) would include it. Gum Arabic, which cannot act as a rigid reinforcement, raised the modulus 1.81-fold even though the GA study reported a decrease in crystallinity [29]. Within the limits of these illustrative estimates, the stiffening by Gum Arabic, and probably by GO and MWCNT, is consistent with a change in the PVA network or in the porosity of the film, although reinforcement cannot be excluded for aligned nanotubes or, under the most favorable assumptions, for GO. The Halpin–Tsai calculation tests only whether rigid reinforcement could account for the observed stiffening. It provides bounds rather than a mechanism and cannot separate physical crosslinking from changes in hydrogen bonding.

## 4. Discussion

### 4.1. Implications for Assessing Chlorine Resistance

The bias identified in this work is not specific to the present membranes. Percent rejection retention is the prevailing metric of chlorine resistance, yet Equation (5) demonstrates that it systematically favors the more selective membrane. Because this bias is inherent to the metric rather than to any particular membrane or exposure condition, it affects comparisons across filler types, loadings and studies alike. Reporting k_R_, or preferably B and the water flux before and after exposure, would enable chlorine stability and selectivity to be assessed independently, in line with the use of A and B to benchmark desalination membranes [47].

### 4.2. Comparison with Polyamide

The unmodified PVA membranes gave k_R_ = 1.8–3.2 after about 4000 mg L^−1^·h at pH 4. Polyamide membranes deteriorate markedly after exposures of the order of 1000 ppm·h [7], and Ortiz–Medina et al. reported a fall in rejection to 82% for a nanotube-free polyamide membrane after 4800 ppm·h [18]. These figures cannot be used to rank the two materials. The polyamide test was carried out at pH ≈ 9, where the hypochlorite ion predominates, whereas the present exposures were at pH 4, where hypochlorous acid predominates, and the membranes also differ in structure and thickness. Nor is the crosslinked PVA network free of aromatic groups: DGEBA contributes bisphenol A units, although it makes up only about 0.7% of the solids in the casting solution (Section 2.1). The comparison is given only as context for the magnitude of k_R_.

### 4.3. Implications for the Protection Mechanism

The GA study attributed the enhanced chlorine resistance to hydrogen bonding between the hydroxyl groups of PVA and Gum Arabic [29], and the MWCNT study to physical crosslinking that was thought to reduce the sites available for chlorine attack [28]. An alternative chemical mechanism is sacrificial protection, whereby reactive groups on the filler are consumed in preference to those of the network [12,13]. If any of these mechanisms were dominant, the modified membranes should have lower k_R_ values than their controls, and the reduction should grow with loading until it levels off. The data address these hypotheses only partially, and the outcome differs between fillers. For GO, the absence of any consistent reduction in k_R_ provides no evidence of net chemical protection under the present conditions. This inference is indirect: a protective effect offset by another change on chlorination, such as greater swelling, cannot be excluded without spectroscopic and swelling measurements on the exposed membranes. For MWCNT, k_R_ was reduced at every loading from 0.02 wt% upwards, without a monotonic trend; this pattern would arise from a chemical mechanism that saturates at very low loading, but equally from a physical one, and the data do not discriminate between the two. For GA, no reduction in k_R_ was detected at 0.7–0.9 wt%; because k_R_ is poorly constrained at an initial rejection of 97–98%, the data neither confirm nor exclude protection at these loadings, but they do not show the growth with loading expected of a protective additive.

The most evident effect of the fillers was an increase in initial rejection, the origin of which is central to the interpretation of the chlorine data. Impermeable fillers can impede transport by increasing the tortuosity of the diffusion path [48,49], but this reduces A and B by the same factor. At a given net driving pressure, J_w_ then decreases in proportion to A (Equation (1)), leaving B/J_w_, and hence the rejection (Equation (3)), unchanged. In the GO study, however, the water flux in the performance tests increased from 66.0 to 91.3 L m^−2^ h^−1^ while the rejection rose from 69.5% to 97.8% (0–0.08 wt% GO; Table S2) [30], implying a 19.5-fold decrease in B/J_w_ together with an increase of about 1.7-fold in the water permeance A. This estimate takes the osmotic pressure of the 3.28 wt% feed as about 25 bar and neglects concentration polarization, which would make the increase larger. The same combination is found for MWCNT (0.08 wt%: B/J_w_ 8.3-fold lower, A about 2.4-fold higher) and for GA (0.9 wt%: 20.7-fold lower and 1.9-fold higher). Tortuosity alone cannot account for this combination, and neither can a change in the thickness of the selective layer, which would also change A and B by the same factor. The combination points instead to a change in the selective material itself, either in the polymer network, for instance in crosslink density or free volume, or through preferential water pathways at the filler–polymer interface; the present data cannot distinguish these possibilities. The GO study described GO as acting as a physical crosslinker [30], and strong hydrogen bonding between GO and PVA is well-documented [27]. Swelling, crystallinity or free-volume measurements would be needed to confirm such a change.

The mechanical and surface data are consistent with this interpretation but do not go further. The selectivity gain rises with stiffness (Figure S1a), the stiffening by MWCNT exceeds the illustrative estimate for in-plane random reinforcement though not the aligned upper bound, and Gum Arabic, which cannot act as a rigid filler, stiffened the membrane even though the GA study reported a decrease in crystallinity [29] (Section 3.4). For Gum Arabic, the stiffening is therefore not due to increased crystallinity; it is consistent with a change in the PVA network or with the lower surface porosity reported in [29]. The data cannot distinguish additional physical crosslinking from a reorganized hydrogen-bond network, and for aligned nanotubes, or for GO under the most favorable assumptions, they cannot exclude reinforcement by the filler itself. Surface properties show no relationship with either selectivity or k_R_.

Whatever its origin, a larger gain in selectivity was not associated with a smaller k_R_ (Figure S1b). The same reasoning applies to hydrogen bonding, which the GA study invoked to explain both the higher rejection and the improved chlorine resistance [29]. Hydrogen bonds between the filler and the hydroxyl groups of PVA act on the membrane before exposure: by restricting chain mobility and reducing free volume, they can raise the selectivity of the network and so raise R_b_. k_R_, in contrast, measures the relative change in salt passage caused by exposure. For hydrogen bonding to lower k_R_, it would have to slow the reaction of chlorine with the network, for example, by limiting the access of hypochlorous acid to reactive sites, or make salt transport less sensitive to a given extent of reaction, for example, by restraining the swelling of the oxidized network. A tighter initial structure does not necessarily produce either effect. The hydrogen-bonded hydroxyl groups remain in the network and are presumably still open to oxidation, and hypochlorous acid, a small neutral molecule, is likely to be excluded far less effectively than hydrated ions. A filler can therefore raise R_b_ substantially while leaving k_R_ unchanged, as observed for GO. Conversely, the reductions in k_R_ found for MWCNT and for GA at low loading are not explained by the gain in selectivity alone and need an additional cause. The clearest case is GA at 0.1 wt%, where k_R_ fell to 0.55 times the control value without any gain in selectivity.

Although rejection data alone cannot identify the site of chlorine attack, the chemistry of the constituents suggests several possibilities. At pH 4, free chlorine is present mainly as hypochlorous acid, and hypochlorite in acidic media is known to oxidize secondary alcohols to ketones [50], rendering the secondary hydroxyl groups of PVA a plausible target. Ye et al. observed only minor degradation of dissolved PVA by chlorine alone [51], albeit under considerably milder conditions than a 2 h exposure to 2000 mg L^−1^ at pH 4. The DGEBA crosslinks, which contain the aromatic rings of bisphenol A, may be less susceptible, given that monomeric bisphenol A diglycidyl ether did not react measurably with free chlorine under drinking-water conditions [52]; that study, however, concerned the free compound at low chlorine concentrations and does not establish the behavior of the crosslinked DGEBA–PVA network at 2000 mg L^−1^ and pH 4. Attack on the polyether segments of Pluronic F-127, or physical relaxation of the swollen selective layer, cannot be excluded. For MWCNT and low-loading GA, restricted swelling, which would limit the penetration of chlorine into the network, is one possible explanation, and the absence of a detectable reduction at high GA loadings may reflect the additional oxidizable polysaccharide. Both are hypotheses that would require swelling, FTIR and XPS measurements before and after exposure. Table 4 summarizes the extent to which each mechanism is consistent with the present results.

### 4.4. Limitations

The main limitations, most of which are noted where they arise above, are as follows. First, k_R_ measures the change in B/J_w_ under the test conditions (if concentration polarization is neglected), not chemical degradation; without the flux after exposure, changes in B, flux, swelling and concentration polarization cannot be separated (Section 2.3). This interpretation also assumes solution–diffusion transport. The low rejections of the controls, the micrometer-scale surface pores produced by Pluronic F-127 [28,29,30] and the close agreement between rejections measured with 2000 mg L^−1^ NaCl and with the 3.28 wt% sea-salt feed suggest that convective flow may contribute; Equation (5) then remains valid as a relation between R_b_, R_a_ and k_R_, but k_R_ may not correspond to a change in B/J_w_ alone. Second, the analysis uses mean rejections, and each series has a single control; the sensitivity analysis shows that the main conclusions hold for uncertainties of up to 2–3 percentage points (Table S4), but it cannot address a systematic difference in a control batch. The spread among the three controls suggests that such differences may be as large as the effects examined, so the MWCNT reduction in particular should be regarded as provisional. Third, every membrane was exposed to one condition, 2000 mg L^−1^ hypochlorite at pH 4 for 2 h. At pH 4, free chlorine is present almost entirely as hypochlorous acid, whereas the hypochlorite ion predominates above pH 7.5 [53], and for polyamide both the extent and the nature of degradation at a given dose depend on pH and concentration [23,54]. The secondary hydroxyl groups of PVA, the ether linkages formed with DGEBA and the polyether segments of Pluronic F-127 may likewise respond differently to these variables. The values of k_R_, and possibly the ranking of the fillers, could therefore differ at neutral pH, at lower concentrations or after prolonged exposure, and the conclusions on individual fillers apply only to short, high-concentration exposure under acidic conditions. The bias of rejection retention does not share this restriction, because Equation (5) holds for any value of k_R_. Finally, the mechanisms discussed in Section 4.3 rest on rejection trends and on the properties of the unexposed membranes, and remain to be tested by FTIR, XPS, swelling and residual-chlorine measurements.

## 5. Conclusions

This work is a methodological reanalysis of published chlorination data for substrate-free PVA reverse osmosis membranes modified with MWCNT, Gum Arabic or graphene oxide; no new membranes or experiments are involved. It quantifies how percent rejection retention depends on the initial rejection and compares the apparent chlorine resistance indicated by this metric with the observed change in salt passage. The analysis relies on a single chlorine dose and pH, on mean rejection values, and on a salt-passage ratio that is not a direct measure of degradation, and its conclusions should be read in that light.

Percent rejection retention is strongly biased toward membranes with high initial rejection: for an identical k_R_ of 3, the rejection loss decreases from 38% to 4% as the initial rejection increases from 69% to 98%. All three fillers reduced the relative rejection loss by roughly an order of magnitude at their best-performing loadings. Under a sequential counterfactual decomposition, however, most or all of this improvement for GO and Gum Arabic (97–111% under order A, the more robust of the two orders) is attributable to the higher initial rejection rather than to a smaller change in salt passage, whereas for MWCNT, the attributed share depends on the decomposition order (28–83%).

In terms of k_R_, the three fillers behaved differently. Graphene oxide produced no consistent reduction across loadings (3.11 ± 0.48 versus 3.20 for its control); MWCNT produced a moderate reduction already at 0.02 wt% (1.16–1.49 versus 1.76); and Gum Arabic reduced k_R_ at 0.1–0.5 wt% (1.12–1.34 versus 2.03) but no reduction was detected at 0.7–0.9 wt%, where k_R_ is poorly constrained. The reductions for Gum Arabic and MWCNT remained probable (probability ≥ 0.95) for uncertainties in the mean rejections of up to about 3 and 2 percentage points, respectively, the MWCNT reduction only marginally and with a size comparable to the spread among the controls, while the GO result did not depend on the assumed uncertainty. The data provide no evidence of net chemical protection by GO, but they can neither prove nor exclude any specific chemical mechanism. At their best-performing loadings, the main effect of GO and Gum Arabic was an increase in selectivity, which tortuosity alone cannot explain; for MWCNT, a reduction in k_R_ also contributed.

Mechanical and surface data from the source studies are consistent with this picture, but the evidence is exploratory. The selectivity gain increased with stiffness (ρ = 0.66) and was unrelated to contact angle or roughness; these correlations do not distinguish physical crosslinking, changes in hydrogen bonding, reduced porosity and, for GO and aligned MWCNT, reinforcement by the filler. Higher selectivity, including that produced by hydrogen bonding, raises the initial rejection without necessarily reducing the change in salt passage caused by chlorine, so the moderate reductions in k_R_ found for MWCNT and low-loading Gum Arabic require a separate explanation.

Future work should measure both flux and rejection over a range of chlorine doses and pH values so that B/J_w_, or B after correction for concentration polarization, can be estimated more directly, employ replicate membranes, and use FTIR, XPS and swelling measurements to identify the components of the network affected by chlorine. More broadly, the salt-passage ratio and decomposition approach described here can be applied to published data on other chlorine-resistant membranes to separate the effect of the initial rejection from the change in salt passage.

## Figures and Tables

**Table 1:** Membranes considered in this work.

Study	Filler	Loadings (wt% in Casting Solution)	Crosslinker	Best-Performing Loadings
Falath et al. [28]	MWCNT	0, 0.02, 0.04, 0.06, 0.08, 0.1	DGEBA, 0.16 wt%	0.08–0.1 wt%
Falath et al. [29]	Gum Arabic	0, 0.1, 0.3, 0.5, 0.7, 0.9	DGEBA, 0.16 wt%	0.7–0.9 wt%
Falath [30]	Graphene oxide	0, 0.02, 0.04, 0.06, 0.08, 0.1	DGEBA, 0.16 wt%	0.08–0.1 wt%

All membranes contained 6 wt% Pluronic F-127 and were tested in a dead-end stirred cell; the performance tests were run at 5.52 MPa (55.2 bar).

**Table 2:** Rejection before (R_b_) and after (R_a_) chlorination, relative rejection loss D, observed salt-passage ratio k_R_ and share of the improvement in D attributed to the higher initial rejection.

Series	Loading (wt%)	R_b_ (%)	R_a_ (%)	D (%)	k_R_ (95% Interval)	Share Attributed to Initial Rejection, Order A/B (%)
MWCNT [28]	0 (control)	54.0	40.0	25.9	1.76 (1.71–1.82)	—
	0.02	74.2	71.3	3.9	1.16 (1.11–1.20)	43/13
	0.04	78.1	73.2	6.3	1.31 (1.25–1.36)	59/31
	0.06	84.2	78.1	7.2	1.49 (1.42–1.57)	81/60
	0.08	91.3	89.3	2.2	1.26 (1.15–1.38)	83/35
	0.10	89.2	87.2	2.2	1.21 (1.12–1.31)	77/28
Gum Arabic [29]	0 (control)	69.0	52.3	24.2	2.03 (1.96–2.10)	—
	0.1	68.2	65.7	3.7	1.12 (1.08–1.16)	−2/0
	0.3	78.1	74.5	4.6	1.22 (1.17–1.27)	30/9
	0.5	86.0	82.1	4.5	1.34 (1.26–1.42)	59/25
	0.7	98.3	94.4	4.0	3.43 (2.55–4.91)	111/193
	0.9	97.2	94.2	3.1	2.14 (1.73–2.69)	101/109
Graphene oxide [30]	0 (control)	69	41	40.6	3.20 (3.09–3.32)	—
	0.02	89	70	21.3	3.47 (3.27–3.68)	110/114
	0.04	92	81	12.0	2.70 (2.49–2.93)	89/79
	0.06	95	86	9.5	3.09 (2.76–3.49)	99/96
	0.08	98	95	3.1	2.58 (1.96–3.53)	97/79
	0.10	98	93	5.1	3.69 (2.87–4.97)	102/114

Shares are relative to the control of the same study, under the sequential decomposition of Section 2.4 (order A: initial rejection changed first; order B: k_R_ changed first). Chlorination: 2000 mg L^−1^ hypochlorite solution, pH 4.0, 2 h (nominal 4000 mg L^−1^·h); rejection measured with 2000 mg L^−1^ NaCl.

**Table 3:** Observed increase in Young’s modulus compared with illustrative Halpin–Tsai estimates for rigid-filler reinforcement.

Filler (Loading)	Observed E/E_0_	Estimated E/E_0_, Random Orientation	Estimated E/E_0_, Aligned
GO (0.08 wt%; φ ≈ 0.0023–0.0031)	2.02	1.11–1.51 (3D random)	1.23–2.04 (platelets in film plane)
MWCNT (0.06 wt%; φ ≈ 0.0018–0.0024)	2.30	1.18–2.14 (random in film plane)	1.49–4.03
Gum Arabic (0.9 wt%)	1.81	Not applicable (soluble polymer)	Not applicable

Ranges correspond to E_f_ = 40–250 GPa and α = 300–700 for GO, and E_f_ = 100–1000 GPa and l/d = 300–833 for MWCNT, with Pluronic F-127 either retained or fully extracted with complete densification; E_m_ = 0.2 GPa.

**Table 4:** Proposed chlorine-resistance mechanisms compared with the trends in k_R_.

Mechanism	Expected Trend in k_R_	Observed	Assessment
Chemical protection by hydrogen bonding [29]	Lower than control; may fall with loading or saturate	GO: no consistent reduction; MWCNT: reduction at all loadings from 0.02 wt%; GA: reduction at 0.1–0.5 wt%, none detected at 0.7–0.9 wt% (k_R_ poorly constrained)	No net effect detected for GO; not excluded for MWCNT; not supported at high GA loading
Sacrificial oxidation of filler [12,13]	Lower than control until filler is consumed	As above	Not supported for GO; not excluded for MWCNT or low-loading GA; sacrificial capacity not assessed
Higher selectivity of the network (lower B/J_w_)	No change in k_R_; D falls because R_b_ rises	GO: consistent; GA at 0.7–0.9 wt%: compatible (k_R_ not lower than control, but poorly constrained); MWCNT: partly (k_R_ reduced at all loadings); selectivity gain correlates with stiffness (ρ = 0.66) but not with surface properties	Main contribution for GO and GA at best-performing loadings; partial for MWCNT
Reduced access of chlorine to the network (e.g., lower swelling or physical crosslinking [28])	Lower k_R_, possibly saturating at low loading	MWCNT (all loadings); GA (0.1–0.5 wt%)	Hypothesis; requires swelling data

## Data Availability

The original contributions presented in this study are included in the article and Supplementary Materials (Tables S1–S4 and Code S1). All values were taken from the original laboratory records of the source studies [28,29,30]; Tables S1 and S2 list them with the corresponding figures and pages of those publications. The laboratory records of the averaged values used in this study are available from the corresponding author on request.

## Supplementary Materials

The following supporting information can be downloaded at https://www.mdpi.com/article/10.3390/polym18212796/s1, Figure S1: Relation between properties of the modified membranes, each relative to the control of its own study: (a) selectivity gain, (B/J_w_)0/(B/J_w_), against relative Young’s modulus; (b) relative salt-passage ratio against selectivity gain; (c) relative salt-passage ratio against water contact angle. Spearman ρ and permutation *p*-values over the fifteen modified membranes are given above each panel. The positive correlation in (b) is partly a consequence of the before-exposure salt passage on which both quantities depend (Section 2.6 of the main text). These correlations are exploratory. Property values from Table S2; Table S1: Rejection before and after chlorination for all membranes, with sources (CSV); Table S2: Contact angle, surface roughness, Young’s modulus, flux and rejection of all membranes, with source figures (CSV); Table S3: Sensitivity of the kR comparisons to the assumed uncertainty in the mean rejections. (a) Series means relative to the control; (b) individual membranes; (c) decomposition shares at the optimal loadings. Values are medians with 95% intervals from 20,000 Monte Carlo draws; P is the fraction of draws in which kR (or the series-mean kR) is lower than that of the control, whose rejections were perturbed in the same way (see Section 2.5 of the main text). Normal distributions were truncated to 0–100%; Table S4: Probability that the series-mean kR lies below that of the control as the assumed standard deviation of each mean rejection is increased (normal distributions truncated to 0–100%; 20,000 draws per point). The largest standard deviations at which the probability remains at least 0.95 are 2.0 percentage points for MWCNT and 3.25 percentage points for Gum Arabic at 0.1–0.5 wt%; for graphene oxide the probability stays between 0.43 and 0.56 throughout; Code S1: Python script reproducing Table 2, the property correlations, Table 3, Table S3 and Table S4.

## Abbreviations

The following abbreviations are used in this manuscript:

DGEBA	bisphenol A diglycidyl ether
GA	Gum Arabic
GO	graphene oxide
MWCNT	multi-walled carbon nanotubes
PVA	poly(vinyl alcohol)
RO	reverse osmosis
