# Supplementary material for: Rejection Retention as a Measure of Chlorine Resistance: Initial-Rejection Bias in Filler-Modified Poly(vinyl alcohol) Reverse Osmosis Membranes
Source: Polymers (Basel). 2026 Nov 9;18(21):2796. doi: 10.3390/polym18212796 (PMC13765774; doi:10.3390/polym18212796)
Supplement: Supplementary file 1 [file polymers-18-02796-s001.zip › Supplementary_Materials.pdf]

Supplementary Materials

Rejection Retention as a Measure of Chlorine Resistance: Initial-Rejection Bias in Filler-Modified Poly(vinyl alcohol) Reverse Osmosis Membranes

Wail Falath

Contents: Figure S1; Tables S3 and S4 (summarized here; the full values are generated by Code S1). Tables S1 and S2 and Code S1 are provided as separate files: Table_S1.csv gives the rejections before and after chlorination with their sources, Table_S2.csv gives the contact angle, roughness, Young's modulus, flux and rejection of every membrane with the source figure and page, and Code_S1.py reproduces Table 2, the property correlations, Table 3 and Tables S3 and S4.

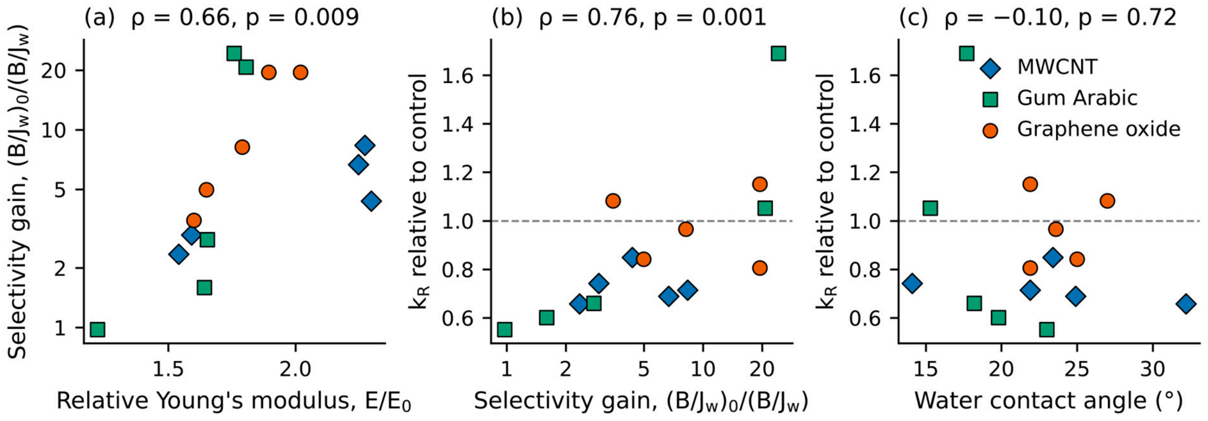

Figure S1. Relation between properties of the modified membranes, each relative to the control of its own study: (a) selectivity gain, $(B/J_w)_0/(B/J_w)$, against relative Young's modulus; (b) relative salt-passage ratio against selectivity gain; (c) relative salt-passage ratio against water contact angle. Spearman ϱ and permutation p-values over the fifteen modified membranes are given above each panel. The positive correlation in (b) is partly a consequence of the before-exposure salt passage on which both quantities depend (Section 2.6 of the main text). These correlations are exploratory. Property values from Table S2.

Table S3. Sensitivity of the k_R comparisons to the assumed uncertainty in the mean rejections. (a) Series means relative to the control; (b) individual membranes; (c) decomposition shares at the optimal loadings. Values are medians with 95% intervals from 20,000 Monte Carlo draws; P is the fraction of draws in which k_R (or the series-mean k_R) is lower than that of the control, whose rejections were perturbed in the same way (see Section 2.5 of the main text). Normal distributions were truncated to 0–100%.

(a) Series-mean k_R divided by control k_R

Series	Loadings	±0.5 pp (uniform)		SD 1 pp (normal)		SD 2 pp (normal)	
		Ratio	P	Ratio	P	Ratio	P
MWCNT	0.02–0.10 wt%	0.73 (0.70–0.76)	1.00	0.73 (0.63–0.86)	1.00	0.75 (0.55–1.03)	0.96
Gum Arabic	0.1–0.5 wt%	0.60 (0.58–0.63)	1.00	0.61 (0.52–0.71)	1.00	0.61 (0.45–0.84)	1.00
Gum Arabic	0.7–0.9 wt%	1.38 (1.12–1.77)	0.00	1.45 (0.75–7.73)	0.16	1.34 (0.43–16.24)	0.33
Graphene oxide	0.02–0.10 wt%	0.98 (0.89–1.09)	0.66	1.03 (0.75–3.06)	0.44	1.06 (0.61–6.42)	0.43

(b) Individual membranes: k_R and probability of lying below the control

Series	Loading (wt%)	±0.5 pp (uniform)		SD 1 pp (normal)		SD 2 pp (normal)	
		k_R	P	k_R	P	k_R	P
MWCNT	0.02	1.16 (1.11–1.20)	1.00	1.16 (1.01–1.33)	1.00	1.16 (0.88–1.53)	0.99
MWCNT	0.04	1.31 (1.25–1.36)	1.00	1.31 (1.12–1.52)	1.00	1.30 (0.97–1.78)	0.94
MWCNT	0.06	1.49 (1.42–1.57)	1.00	1.49 (1.25–1.81)	0.93	1.49 (1.04–2.22)	0.77
MWCNT	0.08	1.26 (1.15–1.38)	1.00	1.26 (0.91–1.75)	0.97	1.26 (0.65–2.57)	0.83
MWCNT	0.1	1.21 (1.12–1.31)	1.00	1.21 (0.93–1.60)	0.99	1.21 (0.71–2.18)	0.89
Gum Arabic	0.1	1.12 (1.08–1.16)	1.00	1.12 (0.99–1.27)	1.00	1.12 (0.87–1.44)	1.00
Gum Arabic	0.3	1.22 (1.17–1.27)	1.00	1.22 (1.05–1.42)	1.00	1.22 (0.90–1.67)	0.99
Gum Arabic	0.5	1.34 (1.26–1.42)	1.00	1.34 (1.09–1.66)	1.00	1.34 (0.88–2.09)	0.95
Gum Arabic	0.7	3.43 (2.56–4.90)	0.00	3.31 (1.37–25.75)	0.17	2.59 (0.57–39.03)	0.38
Gum Arabic	0.9	2.14 (1.73–2.68)	0.35	2.14 (1.05–7.21)	0.45	1.99 (0.49–22.95)	0.51
Graphene oxide	0.02	3.47 (3.27–3.68)	0.01	3.47 (2.81–4.40)	0.27	3.47 (2.32–5.85)	0.38
Graphene oxide	0.04	2.70 (2.50–2.92)	1.00	2.70 (2.05–3.73)	0.84	2.71 (1.60–5.73)	0.69
Graphene oxide	0.06	3.09 (2.76–3.49)	0.66	3.10 (2.09–5.34)	0.55	3.10 (1.50–13.11)	0.53
Graphene oxide	0.08	2.57 (1.97–3.53)	0.88	2.52 (1.05–17.22)	0.66	2.10 (0.39–33.62)	0.68
Graphene oxide	0.1	3.69 (2.88–4.96)	0.20	3.65 (1.68–22.24)	0.40	3.04 (0.84–43.18)	0.52

(c) Share of the improvement attributed to the initial rejection (%) at the optimal loadings

Series	Loading (wt%)	±0.5 pp (uniform)		SD 1 pp (normal)		SD 2 pp (normal)	
		Order A	Order B	Order A	Order B	Order A	Order B
MWCNT	0.08	83 (79 to 87)	35 (21 to 51)	83 (69 to 101)	35 (−13 to 103)	83 (58 to 128)	35 (−53 to 225)
MWCNT	0.1	77 (73 to 82)	28 (16 to 41)	77 (64 to 95)	28 (−10 to 83)	77 (54 to 122)	28 (−40 to 177)
Gum Arabic	0.7	111 (105 to 118)	193 (140 to 258)	110 (91 to 133)	187 (39 to 461)	105 (73 to 150)	143 (−53 to 560)
Gum Arabic	0.9	101 (96 to 107)	109 (74 to 151)	101 (83 to 125)	109 (6 to 332)	99 (67 to 146)	97 (−62 to 501)
Graphene oxide	0.08	97 (93 to 101)	79 (54 to 109)	97 (81 to 112)	77 (3 to 221)	94 (67 to 118)	60 (−48 to 248)
Graphene oxide	0.1	102 (98 to 107)	114 (89 to 142)	102 (86 to 119)	113 (39 to 240)	99 (71 to 126)	95 (−10 to 266)

Table S4. Probability that the series-mean k_R lies below that of the control as the assumed standard deviation of each mean rejection is increased (normal distributions truncated to 0–100%; 20,000 draws per point). The largest standard deviations at which the probability remains at least 0.95 are 2.0 percentage points for MWCNT and 3.25 percentage points for Gum Arabic at 0.1–0.5 wt%; for graphene oxide the probability stays between 0.43 and 0.56 throughout.

SD of each mean rejection (pp)	MWCNT, 0.02–0.10 wt%	Gum Arabic, 0.1–0.5 wt%	Graphene oxide, 0.02–0.10 wt%
0.50	1.000	1.000	0.556
0.75	1.000	1.000	0.479
1.00	1.000	1.000	0.446
1.25	0.999	1.000	0.433
1.50	0.994	1.000	0.430
1.75	0.982	1.000	0.432
2.00	0.962	0.998	0.435
2.25	0.938	0.995	0.437
2.50	0.909	0.990	0.440
2.75	0.878	0.981	0.441
3.00	0.845	0.971	0.441

SD of each mean rejection (pp)	MWCNT, 0.02–0.10 wt%	Gum Arabic, 0.1–0.5 wt%	Graphene oxide, 0.02–0.10 wt%
3.25	0.816	0.958	0.443
3.50	0.788	0.943	0.444
3.75	0.760	0.929	0.443
4.00	0.736	0.911	0.444
